# Application of ultrasound-guided medical thoracoscopy in patients with small amounts or without pleural effusion

**DOI:** 10.1186/s12890-024-02855-8

**Published:** 2024-01-19

**Authors:** Linhui Yang, Kaige Wang, Wang Hou, Dan Liu, Weimin Li

**Affiliations:** 1 Department of Respiratory and Critical Care Medicine, West China Hospital, Sichuan University, Chengdu, Sichuan China; 2 State Key Laboratory of Respiratory Health and Multimorbidity, West China Hospital, Sichuan University, Chengdu, Sichuan China

**Keywords:** Ultrasound, Medical thoracoscopy, Pleural disease

## Abstract

**Background:**

Pleural disease is a common clinical condition, and some patients present with a small amount of pleural effusion or no pleural effusion. It is difficult to diagnose such patients in clinical practice. Medical thoracoscopy is the gold standard for the diagnosis of pleural effusion with unknown origin, and guidelines recommend that pneumothorax should be induced in such patients before medical thoracoscopy examination. However, the process of inducing pneumothorax is tedious and has many complications. Our study was conducted to clarify the value of thoracic ultrasound combined with medical thoracoscopy in patients with small amounts or without pleural effusion to simplify the process of medical thoracoscopy examination.

**Methods:**

In this retrospective study, we included patients who were assigned to complete medical thoracoscopy. Successful completion of medical thoracoscopy in patients was regarded as letting the endoscope get into the pleural cavity and completion of the biopsy. Finally, we analyzed the value of preoperative ultrasound in patients without or with small amounts of pleural effusion.

**Results:**

Seventy-two patients were finally included in the study. Among them, 68 patients who underwent ultrasound positioning of the access site successfully completed the examination and four patients failed the examination. Fifty-one cases showed no fluid sonolucent area at the access site, of which 48 cases had pleural sliding signs at the access site, and 47 patients successfully completed the examination; 3 cases without pleural sliding signs at the access site failed to complete thoracoscopy. In 21 cases, the fluid sonolucent area was selected as the access site, and all of them successfully completed thoracoscopy.

**Conclusion:**

Medical thoracoscopy is one of the methods to confirm the diagnosis in patients with pleural disease with small amounts or without pleural effusion. The application of thoracic ultrasound before medical thoracoscopy can be used for the selection of the access site. It is possible to replace pneumothorax induction before medical thoracoscopy.

**Supplementary Information:**

The online version contains supplementary material available at 10.1186/s12890-024-02855-8.

## Introduction

Pleural disease, as a common disease, can be either primary, secondary to other diseases or a part of systemic disease. The incidence of pleural disease and its associated diseases (e.g., chronic lung disease, tumors, heart failure, cirrhosis, etc.) has increased significantly worldwide [1]. In a recent systematic review and meta-analysis, medical thoracoscopy demonstrates high diagnostic efficacy, with the area under the summary receiver operating characteristic curve of 0.97 [2]. In addition, thoracoscopy is a safe method of examination [3, 4], and the incidence of operation-related complications is 4% [5]. Therefore, medical thoracoscopy is a more ideal examination to diagnose pleural disease.

Some patients with pleural disease have little or no pleural effusion. Confirming the diagnosis of these patients is very difficult. Guidelines recommend that pneumothorax should be induced in such patients before medical thoracoscopy examination, which is the gold standard for the diagnosis of pleural effusion with unknown origin [6]. However, the process of inducing pneumothorax is tedious and has many complications. In recent years, the introduction and widespread use of thoracic ultrasound have revolutionised the management of pleural diseases [7]. An observational study showed that ultrasound-guided thoracentesis can reduce the risk of pneumothorax by 19% [8]. In addition, thoracic ultrasound is more sensitive than X-ray chest radiographs [8] in detecting pleural effusions and can simultaneously identify isolated pleural effusions and distinguish pleural effusions from pleural thickening. Andrew RL et al. found that thoracic ultrasound prior to medical thoracoscopy improves pleural access and predicts fibrous septation, and thoracic ultrasound can be used to reduce extra procedures and the need for artificial pneumothoraces in patients with pleural effusion (at least 3 cm depth) [9]. However, whether artificial pneumothorax can be replaced by preoperative thoracic ultrasound guidance in patients with small amounts (less than 3 cm depth) or without pleural effusion remains uncertain. In addition, thoracentesis and pleural biopsy are difficult in these patients and the risk of complications (pneumothorax, lung injury) is extremely high. Therefore, this study intends to clarify the application of thoracic ultrasound to locate the access site before medical thoracoscopy operation in patients with small amounts (less than 3 cm depth) or without pleural effusion.

## Methods

We screened 342 patients who underwent medical thoracoscopy at West China Hospital of Sichuan University between July 2018 and June 2021. And all 342 individuals underwent preoperative chest computed tomography (CT)scans, and the initial diagnoses derived from these scans are presented in the Supplemental Table 1. This study was approved by the Ethics Committee of West China Hospital of Sichuan University. Informed consent was waived because this study was retrospective. In this study, we defined a small amount of pleural effusion as pleural fluid with a deepest depth of less than 3 cm as indicated by thoracic ultrasound when the patient was in a sitting position and defined the absence of pleural effusion as the absence of pleural fluid present on both CT scan and ultrasound. And confirmation of the access site was performed prior to the medical thoracoscopy operation, namely, when the patient was prepared for sterilization in lateral decubitus. In other words, the patient was required to undergo two thoracic ultrasound evaluations, one in the sitting position to assess the volume of pleural fluid and another in the lateral position to assess the access site. In addition, we defined a successful medical thoracoscopic operation as the successful entry of the thoracoscope into the pleural cavity and the successful extraction of pleural fluid/acquisition of pleural tissue. Inclusion criteria: (1) Adults aged 18 years or older who underwent thoracoscopy at West China Hospital of Sichuan University from 2018 to 2021; (2) the depth of pleural effusion was ≤ 3 cm as indicated by chest ultrasonography; (3) clinically relevant data (such as the amount of pleural effusion, thoracoscopic biopsy results, etc.) were complete and not missing. The exclusion criteria were as follows: (1) no thoracic ultrasound examination 7 days before the operation; (2) diffuse pleural adhesions; (3) abnormal coagulation system; (4) consciousness disorders or psychiatric diseases that prevented normal communication; and (5) combined cardiac, hepatic, renal and other organic insufficiencies (6). poor general condition, unable to tolerate medical thoracoscopy operation. The patients included in this study were examined with the need of lying on the healthy side under local anesthesia. In addition, we used the medical thoracoscope whose size is LTF-240, semi-rigid and O.D. 7 mm.

We retrospectively collected clinical information on patients’ sex, age, disease duration, nature of pleural effusion, chest CT imaging, ultrasound performance at the access site, thoracoscopic performance of the lesion and biopsy results and complications of the operation. We analysed the role of different ultrasound manifestations of patients in guiding the selection of thoracoscopic access sites.

## Results

We finally included 72 patients for analysis. Among them, male patients and female patients each accounted for 50%. The mean age of the included patients was 54.5 years. In addition, 46 patients had pleural lesions on the right side of the chest, and 26 patients had pleural lesions on the left side. CT scans of the chest in these patients showed the presence of pleural effusion in 59 patients, pleural thickening in 43 patients, and pleural nodules in 10 patients (Table 1).

**Table 1:** Clinical characteristics of enrolled patients

Demographic Data	Patients, No. (%)
Male	36 (50)
Age (y), mean ± SD	54.5 ± 14.5
Location of pleural lesions	
Right side	46 (63.9)
Left side	26 (36.1)
Duration from onset to examination, median (Q1, Q3)	60.0 (20.0, 203.2)
The features of pleural effusion	
Exudative pleural effusion	59 (81.9)
CT manifestations	
pleural effusion	59 (81.9)
Pleural thickening	43 (59.7)
pleural nodules	10 (13.9)

**Fig. 1:**
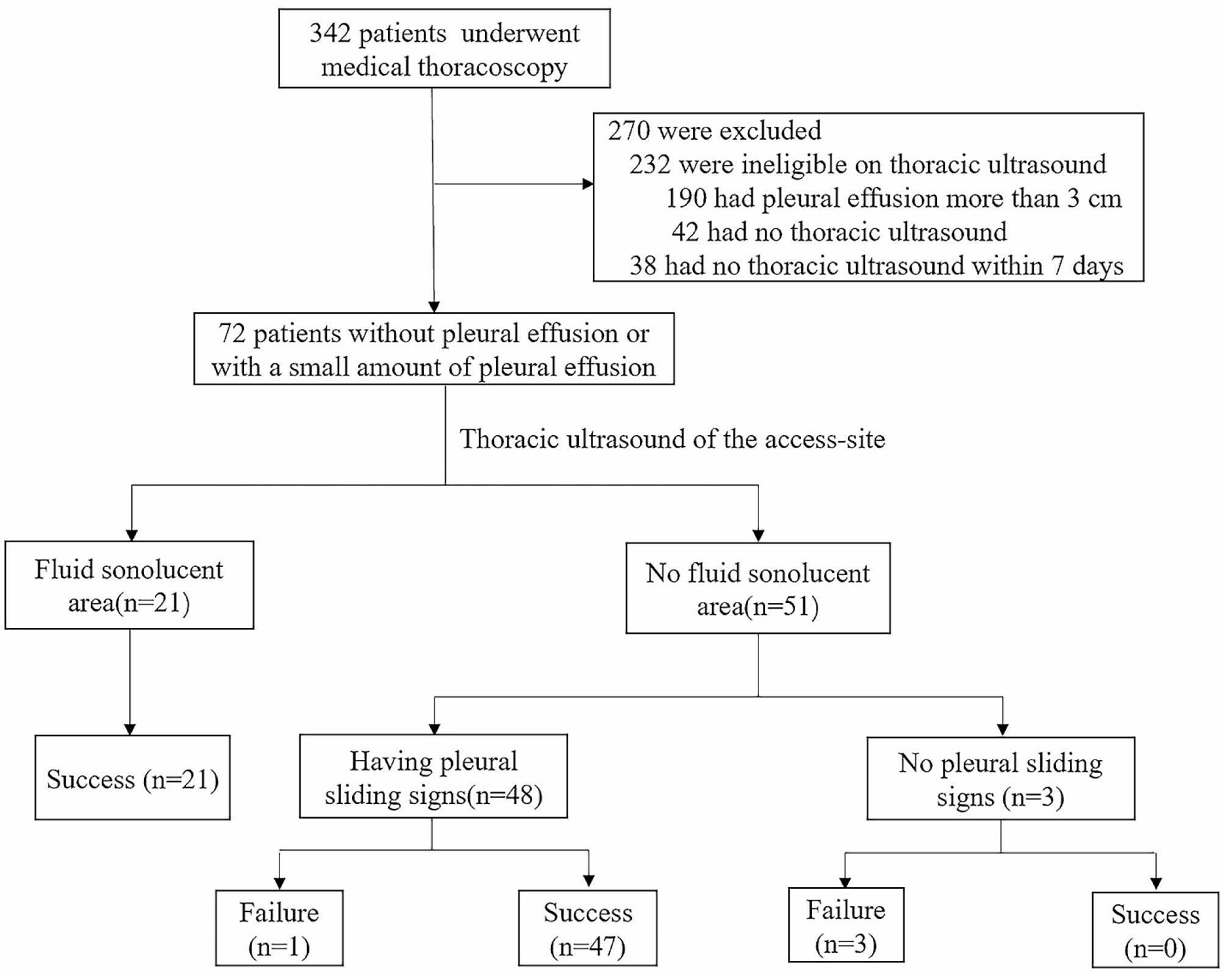
Flow chart of patient screening and grouping

We performed a classification of fifty-one patients whose ultrasound suggested no fluid sonolucent area at the access site, and they were divided into two groups according to the presence or absence of pleural sliding signs on ultrasound. Among them, forty-eight had pleural sliding signs, which indicated that there was no pleural adhesion at the access site on thoracic ultrasound, and forty-seven patients successfully completed medical thoracoscopy. Three patients had ultrasound suggestive of no pleural sliding sign at the access site, and they all failed to complete thoracoscopy because of pleural adhesions. Twenty-one patients who were selected for the fluid sonolucent area as the access site completed medical thoracoscopic operation successfully (Fig. 1).

**Fig. 2:**
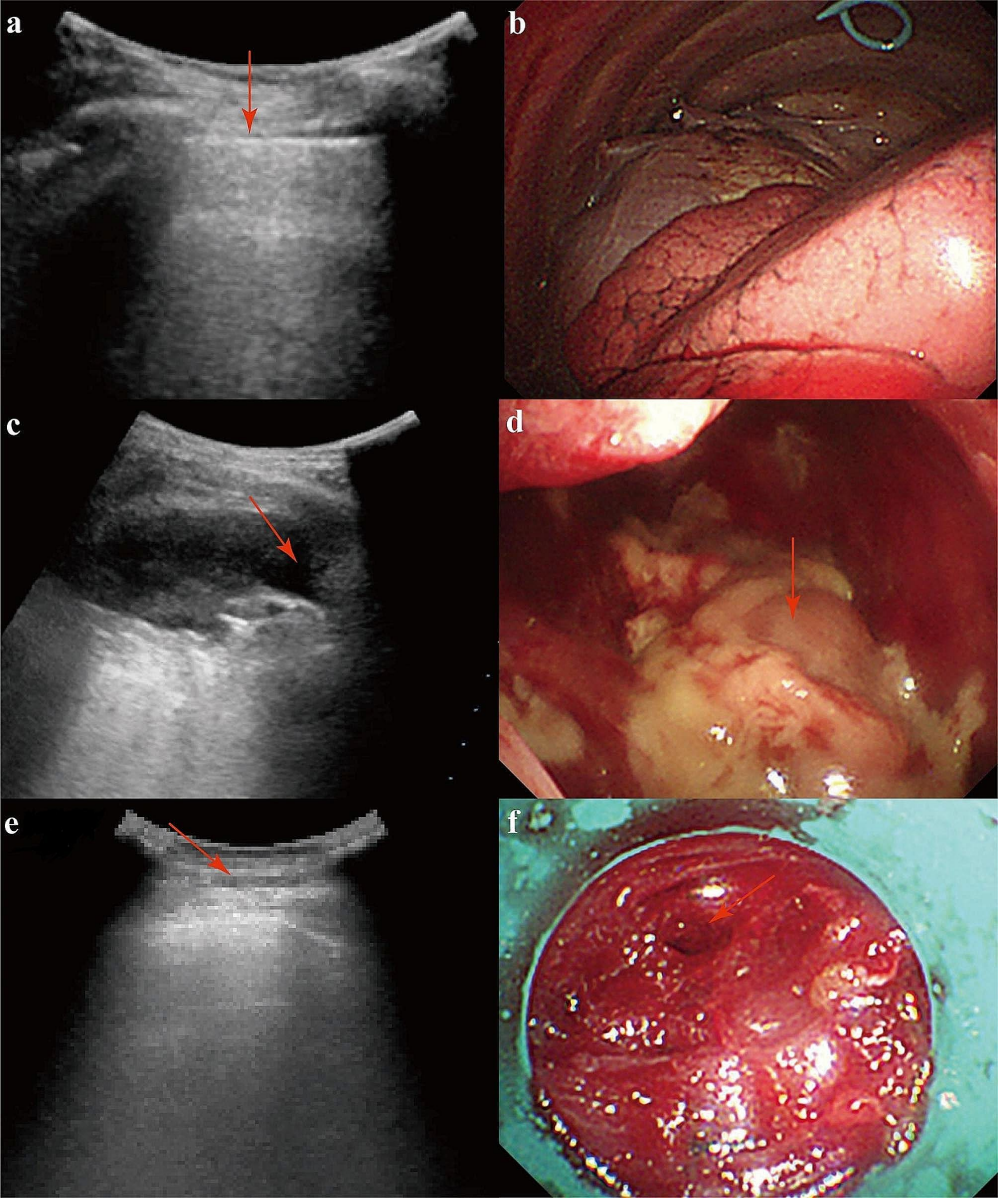
Ultrasound and thoracoscopic presentation of typical patients. (**a**) Ultrasound presentation in a patient with a pleural sliding sign (red arrow). (**b**) Thoracoscopic manifestation of the patient is fully exposed and without pleural adhesions. (**c**) Ultrasound presentation in a patient with a fluid sonolucent area (red arrow). (**d**) Thoracoscopic manifestation of the patient with encapsulated empyema (red arrow). (**e**) Ultrasound presentation in a patient showed thickened pleura (red arrow) without pleural sliding sign. (**f**) Thoracoscopic manifestation of the patient with severe adhesions in the pleural cavity. There was visible injury to the lung (red arrow)

We summarized the thoracoscopic manifestations of 68 patients with successful operations (Table 2). Of the patients who had approach at the pleural sliding sign (Fig. 2a), thoracoscopy suggested no significant pleural adhesions (Fig. 2b) in 22 patients, pleural thickening in 30 patients, and pleural nodules in 10 patients. Meanwhile, of the patients who had access to the fluid sonolucent area (Fig. 2c), thoracoscopy examination suggested no significant pleural adhesions (Fig. 2d) in 3 patients, pleural thickening in 13 patients, and pleural nodules in no patient. In addition, of the patients who were successfully examined, four had lung injury, and one had a pleural reaction. Of the four patients who failed the operation, three had chest ultrasound suggestive of no pleural sliding sign. In one of these patients, the ultrasound presentation suggested that he had thickened pleura, and no significant pleural sliding was seen. This patient had a failed thoracoscopy and a lung injury (Fig. 2e and f).

**Table 2:** Thoracoscopic manifestations and complications of patients who completed the examination

	No fluid sonolucent area at access-site (47 cases) n, (%)	Fluid sonolucent area at access-site (21 cases) n, (%)	Total (68 cases) n, (%)
Pleural adhesions			
None	22 (46.8)	3 (14.3)	25 (36.8)
Single	15 (31.9)	8 (38.1)	23 (33.8)
Multiple	10 (21.3)	10 (47.6)	20 (29.4)
Pleural adhesions at access-site	2 (4.3)	5 (23.8)	7 (10.3)
Pleural thickening	30 (63.8)	13 (62.9)	43 (63.2)
Pleural nodules	10 (21.3)	0 (0.0)	10 (14.7)
Volume of pleural effusion(ml), median (Q1, Q3)	100(22.5,200)	120(50,200)	100(50,200)
Surgical complications			
Lung injury	3 (6.4)	1 (4.8)	4 (5.9)
Thoracic hemorrhage(>20 ml)	0 (0.0)	0 (0.0)	0 (0.0)
Pleural reaction Incision infection	1 (2.1)	0 (0.0)	1 (1.5)

In addition, the pathological results of pleural tissue in 68 patients with successful thoracoscopy are presented in Table 3. Sixty-two patients (91.2%) were confirmed by the operation. The biopsy results were neoplastic in 23 patients, of which 14 patients had lung cancer and 5 patients had malignant mesothelioma. Nineteen patients had pathological biopsies suggestive of tuberculous pleurisy. Eleven patients had pleural infections. Nine patients had other pleural diseases, such as pleural plaque and pleural sarcoidosis. Six patients had nonspecific chronic inflammation on pleural biopsy. There were two patients with pleural disease with a small amount of pleural effusion and no pleural effusion who received medical thoracoscopy.

**Table 3:** Histologic results

Histologic results	n, (%)
Malignant tumor	
Lung cancer	14, (20.6)
Malignant mesothelioma	5, (7.4)
Other tumors	4, (5.9)
Tuberculous pleurisy	19, (27.9)
Pleural infection	11, (16.2)
Nonspecific chronic pleurisy	6, (8.8)
Other pleural diseases	9, (13.2)

### Case 1

**Fig. 3:**
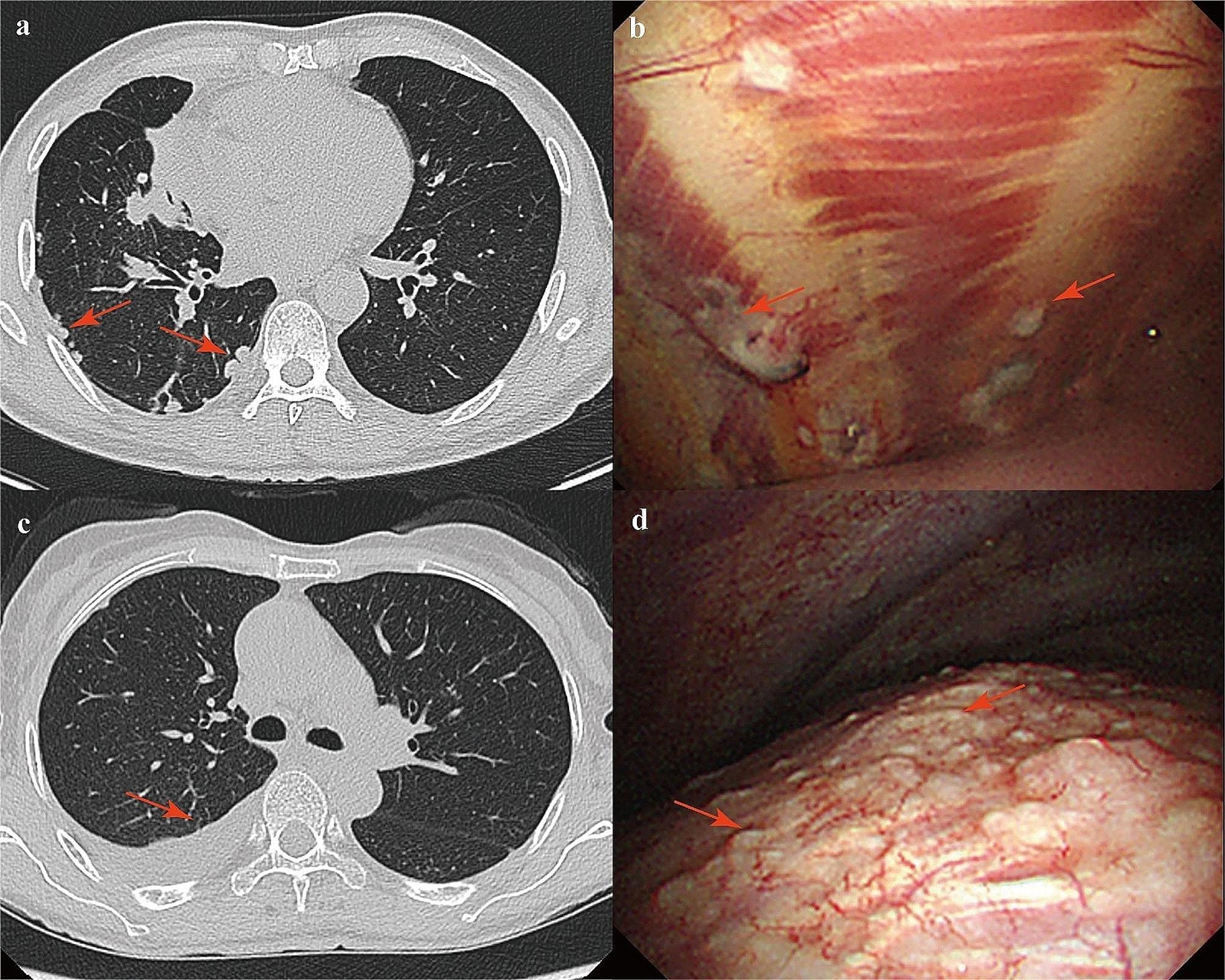
Thoracic CT scan and thoracoscopic presentation of typical patients. (**a**) Thoracic CT scan presentation in the patient with multiple nodules. (**b**) Thoracoscopic manifestation in the patient with multiple pleural nodules. He was diagnosed with pleural metastasis from lung cancer. (**c**) Thoracic CT scan of a patient with tuberculous pleurisy showed a small amount of effusion in the right pleural cavity. (**d**) Thoracoscopic manifestation in the patient showed multiple nodules in the diaphragm pleura, and he was diagnosed with tuberculous pleurisy

This patient is a 71-year-old male admitted to the hospital with intermittent cough and chest pain for 1 month. After admission, the patient underwent a CT scan of the chest suggesting right middle lobe atelectasis, multiple solid nodular shadows in the right lung and subpleural area, and uneven thickening of the pleura (Fig. 3a). Thoracic ultrasound suggested pleural sliding signs at the access site. The patient underwent further thoracoscopy, which revealed diffuse nodules in the visceral pleura and parietal pleura and no pleural effusion (Fig. 3b). Under direct thoracoscopic view, we clamped the multiple lesions for pathological biopsy. The biopsy results suggested that tumor cells were detected, and the immunohistochemical results supported adenocarcinoma of pulmonary origin.

### Case 2

This is a 54-year-old female patient who was admitted with abdominal distension for 7 months and pleural effusion for 6 months. The patient had previously undergone laparoscopic exploration with biopsy at an outside hospital for abdominal distention. Intraoperatively, hemorrhagic ascites was observed, and a large number of diffusely distributed small nodules were observed in the greater omentum and abdominal wall. Postoperative pathology suggested granulomatous inflammation with negative antacid staining, and no DNA fragments of Mycobacterium tuberculosis were detected. After admission, the patient completed relevant examinations: routine examination of pleural fluid indicated a significant increase in nucleated cells and erythrocytes, with single nucleated cells accounting for 85% and multiple nucleated cells accounting for 15%, and no mesothelial cells were detected; pleural fluid smear and culture did not detect bacteria, fungi, or antacid bacilli; pleural fluid tumor markers indicated a serum glycoconjugate antigen 125 value of 1283 U/ml; pleural fluid tuberculosis antibody, pleural fluid tuberculosis DNA, and rifampicin resistance gene tests were all negative. The patient’s chest CT scan prior to thoracoscopy suggested a small amount of fluid in the right pleural cavity (Fig. 3c). Considering the patient’s complicated condition, long duration of disease, and recurrent plural effusion, we performed ultrasound-guided thoracoscopy for the patient whose access site showed a fluid sonolucent area (depth = 2 cm), and medical thoracoscopy revealed multiple nodules in the mural pleura and diaphragmatic pleura (Fig. 3d). Pleural biopsy results suggested granulomatous inflammation, and no tuberculosis DNA and negative antacid staining were detected. Finally, combined with a strong positive tuberculin skin test and thoracoscopic biopsy results, tuberculous pleurisy and tuberculous peritonitis were diagnosed. She improved after diagnostic anti-tuberculosis treatment with rifampicin, isoniazid, ethambutol and pyrazinamide.

## Discussion

We retrospectively analysed the application of ultrasound-guided medical thoracoscopy in patients with small amounts (less than 3 cm) or without pleural effusion. As a result, 94.4% of the patients successfully completed the operation without artificial pneumothorax. A total of 91.2% of the patients were diagnosed by the operation. A total of 5.9% of the patients presented with operation-correlated lung stab injuries. The diagnostic efficacy and safety are consistent with literature reports [6]. The etiology of pleural diseases in this study is consistent with the other literature [10].

Medical thoracoscopy has a high diagnostic value for malignant pleural effusion and tuberculosis [11–14]. It is also a safe method for pleural disease [6]. BTS guidelines recommend that pneumothorax should be induced in patients with small or no pleural effusion before medical thoracoscopy operation. However, complications of pneumothorax induction are common, including hemorrhage because of damage to the blood vessels and pleural reaction. The most serious complication is air embolism caused by air entering the pulmonary vein [15]. Moreover, the procedure of artificial pneumothorax is complex, which not only increases the patient’s pain but also increases medical expenses. There is no randomized controlled study confirming the necessity and alternative treatment measures of artificial pneumothorax.

Thoracic ultrasound can well explore the situation of the pleural cavity, such as whether adhesions occur, the presence of pleural effusion, the amount of pleural effusion and the nature of pleural effusion (encapsulated, nonencapsulated) [16]. Some studies have demonstrated that ultrasound can guide medical thoracoscopy into the thoracic cavity and replace the VATS approach when operated on by an experienced physician, even in the complete absence of pleural effusion [17]. John P Corcoran excluded pleural adhesions by the presence of lung sliding in thoracic ultrasound. In his study, pneumothorax was induced through ultrasound guidance prior to thoracoscopy in patients without pleural effusion, and 87.0% of these procedures were successful [18], which means thoracic ultrasound may improve clinic diagnosis. Therefore, thoracic ultrasound is increasingly used to assist clinicians with cutting-needle biopsies [19] and thoracoscopies [17]. In conjunction with the superiority of thoracoscopy mentioned in the previous literature, this study used preoperative thoracic ultrasound for determining the access site of medical thoracoscopic to investigate its application in patients with no or small amounts (less than 3 cm depth) of pleural effusions.

Our results show that thoracic ultrasound can replace pneumothorax induction before medical thoracoscopy in patients who develop pleural disease without pleural effusion or with a small amount of pleural effusion. In contrast to previous studies, our study also clearly presents the ultrasound performance at the access site that favors the completion of medical thoracoscopy. All patients who were selected for the fluid sonolucent area as the access site completed medical thoracoscopic operation successfully. It is suggested that the local pleural effusion area is a good choice for the access site, which is consistent with previous reports [6]. As has been reported in the previous literature, the sliding sign facilitates the clinician in identifying thoracic adhesions [20, 21, 22], which in turn helps the surgeon in the selection of the surgical procedure and in reducing surgical complications. Forty-eight patients had pleural sliding signs, indicating no pleural adhesion at the access site. Only one patient failed to complete the operation due to pleural adhesions.

## Conclusion

Patients with small amounts or without pleural effusion can also be safely examined with medical thoracoscopy. Selecting the area with the pleural sliding sign or fluid sonolucent area as the access site can effectively avoid areas of pleural adhesions and facilitate complete medical thoracoscopy operation. Thoracic ultrasound can replace pneumothorax induction before medical thoracoscopy.

### Electronic supplementary material

Below is the link to the electronic supplementary material.

Supplementary Material 1

## Data Availability

No datasets were generated or analysed during the current study.

## Abbreviations

CT: Computed tomography
DNA: Deoxyribonucleic acid
VATS: Video-assisted thoracoscopic surgery
