# Supplementary material for: Application of ultrasound-guided medical thoracoscopy in patients with small amounts or without pleural effusion
Source: BMC Pulm Med. 2024 Jan 19;24:42. doi: 10.1186/s12890-024-02855-8 (PMC10797946; doi:10.1186/s12890-024-02855-8)
Supplement: Supplementary file 1 — Supplementary Material 1 [file 12890_2024_2855_MOESM1_ESM.docx]

Supplemental Table1 Primary Diagnosis According to CT scan

| Histologic results | n (%) |
| --- | --- |
| Tumor | 20 (27.8) |
| Tuberculous pleurisy | 22 (30.6) |
| Inflammatory pleural fluid | 14 (19.5) |
| Empyema | 8 (11.1) |
| Others | 8 (11.1) |
